# Rash and Fever in a Returned Traveler

**DOI:** 10.5811/cpcem.1911

**Published:** 2024-03-26

**Authors:** Helena Kons, Elliott D. Herron, Zachary S. Pacheco, Erin F. Shufflebarger

**Affiliations:** University of Alabama at Birmingham Heersink School of Medicine, Department of Emergency Medicine, Birmingham, Alabama

**Keywords:** *fever in returned traveler*, *tropical medicine*, *dengue*

## Abstract

**Case Presentation:**

A 21-year-old, otherwise healthy female presented to the emergency department with fever among other nonspecific symptoms after recently returning from Ghana. On physical exam, she had a characteristic upper extremity rash, and a tourniquet test revealed numerous petechiae. The diagnosis of dengue was suspected and subsequently confirmed.

**Discussion:**

Dengue is one of many viral illnesses that should be considered in returning travelers presenting with fever and other nonspecific symptoms. Emergency physicians must keep a broad differential when evaluating fever in returned travelers and prioritize history and physical exam findings to help narrow the diagnosis and provide appropriate management and supportive care while awaiting further confirmatory testing.

CPC-EM Capsule What do we already know about this clinical entity?

*An acute viral febrile illness transmitted by mosquito, dengue is endemic to Southeast Asia, Latin America, and Africa.*

What makes this presentation of disease reportable?

*This nonspecific presentation of fever and rash illustrates the challenge of diagnosing mosquito-borne viruses.*

What is the major impact of the image?

*A “tourniquet test” revealing petechiae helped narrow the diagnosis.*

How might this improve emergency medicine practice?

*It is necessary to keep a broad differential when evaluating fever in returned travelers and to prioritize history and physical exam findings.*

## CASE PRESENTATION

A 21-year-old, otherwise healthy female presented to the emergency department (ED) with fever after recently returning from Ghana. She reported intermittent fever, headache with photophobia, diarrhea, joint pains, and generalized weakness. She also noticed a diffuse, intermittently pruritic rash. While in Ghana, she volunteered at a refugee hospital, ate local street food, and had exposure to local animals including dogs, sheep, and a monkey.

**Image 1.**
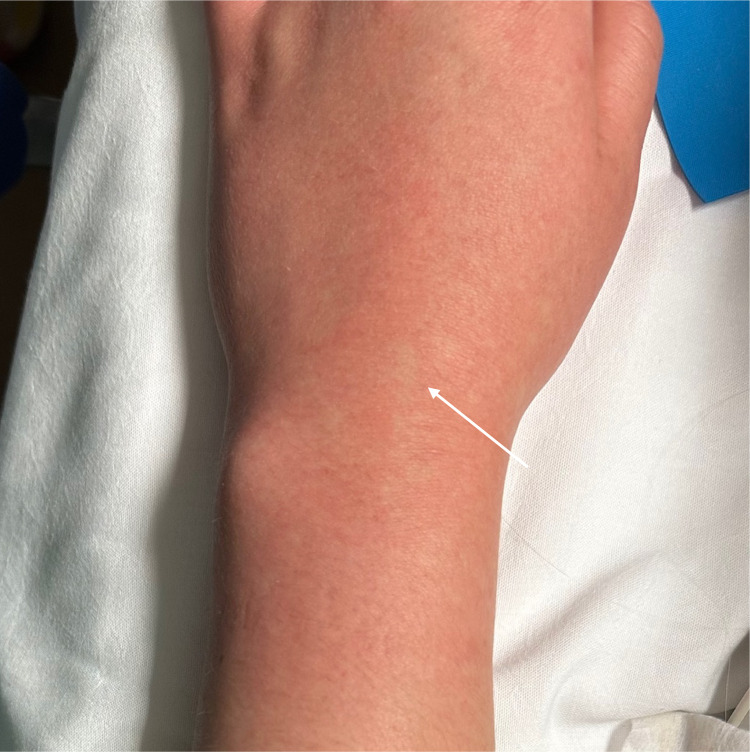
Rash on upper extremity with characteristic confluent erythema and small areas of spared skin (arrow).

**Image 2.**
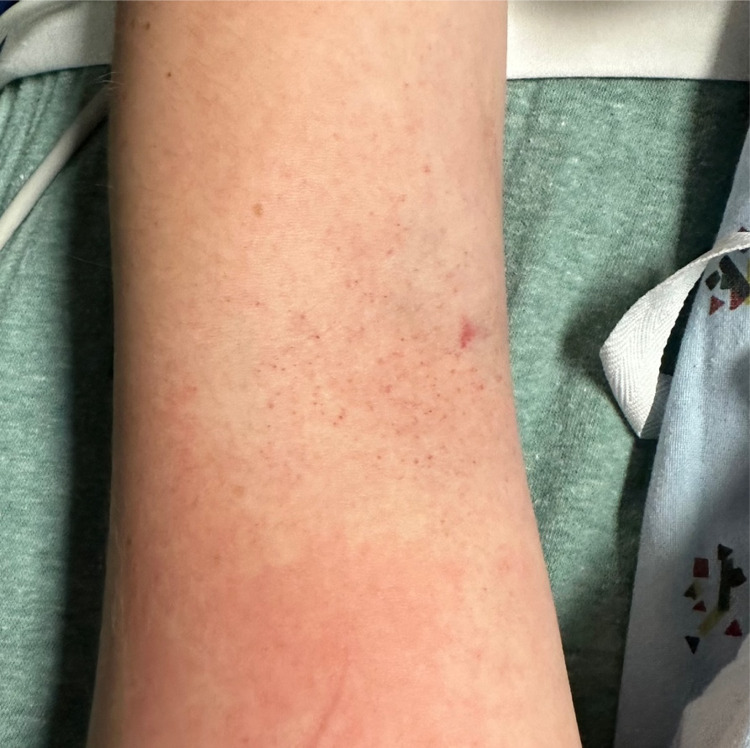
Appreciable petechiae visible in the antecubital fossa after inflating a blood pressure cuff around the upper arm for five minutes at a pressure halfway between the patient’s systolic and diastolic blood pressure. This “tourniquet test” is deemed positive if more than 10 petechiae are present within a square inch of skin, suggesting capillary fragility.^1^

On arrival to the ED, she had a temperature of 39.4° Celsius and was tachycardic at 126 beats per minute. Her other vital signs were within normal limits. Physical exam revealed an uncomfortable-appearing female with a maculopapular rash to the extremities and chest, confluent erythema noted in some areas (Image 1), and scattered papules with some surrounding excoriation around the ankles, which the patient stated were mosquito bites. Initial lab results revealed mildly elevated transaminases with alanine transaminase 58 units per liter (U/L) (reference range 7–52 U/L), aspartate aminotransferase 42 U/L (12–39 U/L), thrombocytopenia with platelets 125.3 × 10^3^ per cubic millimeter (mm^3^) (150–400 × 10^3^/mm^3^), and leukopenia with white blood cells 2.78 × 10^3^/cmm (4–11 × 10^3^/mm^3^). A bedside tourniquet test^1^ was performed (Image 2) to assess for capillary fragility.

The patient received intravenous fluids and acetaminophen for fever and was started on empiric oral doxycycline to cover for tick-related illness prior to admission for observation. Her labs remained stable, and her symptoms, including fever, improved during her 36-hour hospital stay. Approximately one week after discharge from the hospital, both the dengue fever virus antibodies immunoglobulin G and M resulted positive.

## DISCUSSION

Dengue is an acute viral febrile illness transmitted by the *Aedes aegypti* mosquito.^2^ It is endemic to Southeast Asia, Latin America, and Africa.^2^ Within the United States, it remains an uncommon diagnosis, with 814 documented cases reported in 2021.^3^ Dengue commonly presents with nonspecific symptoms including fever, headache, vomiting, transient macular rash, myalgias and arthralgias.^2^ This nonspecific presentation mimics other viral, bacterial, and parasitic illnesses, making it difficult to diagnose in the acute setting. For example, chikungunya symptoms can mirror those of dengue with fever, rash, and myalgias.^4^ Malaria is also mosquito-borne and can present with fever and thrombocytopenia.^4^

Focusing on specific details including region(s) visited, timing of fever relative to incubation period, exposures encountered, symptoms experienced, physical exam findings, and lab results can narrow down the pathogen.^4^ Detection of dengue virus antigens remains the diagnostic gold standard; however, this requires time and specialized equipment.^5^ Therefore, emergency physicians must keep a broad differential when evaluating fever in returned travelers and prioritize history and physical exam findings to help narrow the diagnosis and provide appropriate management while awaiting confirmatory testing.
